# cDNA sequencing improves the detection of *P53 *missense mutations in colorectal cancer

**DOI:** 10.1186/1471-2407-9-278

**Published:** 2009-08-11

**Authors:** Malgorzata Szybka, Magdalena Zakrzewska, Piotr Rieske, Grazyna Pasz-Walczak, Dominika Kulczycka-Wojdala, Izabela Zawlik, Robert Stawski, Dorota Jesionek-Kupnicka, Pawel P Liberski, Radzislaw Kordek

**Affiliations:** 1 Department of Oncological Pathology, Chair of Oncology, Medical University of Lodz, Poland, Paderewskiego 4, 93-509 Lodz, Poland; 2 Department of Molecular Pathology and Neuropathology, Chair of Oncology, Medical University of Lodz, Poland

## Abstract

**Background:**

Recently published data showed discrepancies beteween *P53 *cDNA and DNA sequencing in glioblastomas. We hypothesised that similar discrepancies may be observed in other human cancers.

**Methods:**

To this end, we analyzed 23 colorectal cancers for *P53 *mutations and gene expression using both DNA and cDNA sequencing, real-time PCR and immunohistochemistry.

**Results:**

We found *P53 *gene mutations in 16 cases (15 missense and 1 nonsense). Two of the 15 cases with missense mutations showed alterations based only on cDNA, and not DNA sequencing. Moreover, in 6 of the 15 cases with a cDNA mutation those mutations were difficult to detect in the DNA sequencing, so the results of DNA analysis alone could be misinterpreted if the cDNA sequencing results had not also been available. In all those 15 cases, we observed a higher ratio of the mutated to the wild type template by cDNA analysis, but not by the DNA analysis. Interestingly, a similar overexpression of *P53 *mRNA was present in samples with and without *P53 *mutations.

**Conclusion:**

In terms of colorectal cancer, those discrepancies might be explained under three conditions: 1, overexpression of mutated *P53 *mRNA in cancer cells as compared with normal cells; 2, a higher content of cells without *P53 *mutation (normal cells and cells showing *K-RAS *and/or *APC *but not *P53 *mutation) in samples presenting *P53 *mutation; 3, heterozygous or hemizygous mutations of *P53 *gene. Additionally, for heterozygous mutations unknown mechanism(s) causing selective overproduction of mutated allele should also be considered. Our data offer new clues for studying discrepancy in *P53 *cDNA and DNA sequencing analysis.

## Background

In a recently published study, we showed the prevalence of the mutated *P53 *template by cDNA sequencing and determination of the wild type template by DNA analysis in glioblastomas (GBM). That investigation allowed us to propose three plausible hypotheses to explain those discrepancies: 1, the silencing of wild type mRNA transcription; 2, the degradation of wild type mRNA; 3, the selective overproduction of mutated mRNA [1]. We decided to test if similar discrepancies occur in other human cancers. For this study, colorectal cancer (CC) was selected as a common cancer presenting frequent *P53 *mutations.

The *P53 *gene status was evaluated by means of both cDNA and DNA sequencing, real-time RT-PCR and immunohistochemistry. This comprehensive approach allowed us to go toward an explanation of divergent results observed following cDNA and DNA *P53 *sequencing. Our investigation also sheds new light on the discrepancies between immunohistochemical and molecular analyses of *P53 *in CC.

## Methods

### Tumor samples

Resected specimens from 23 patients with colorectal cancer who underwent resection in the Clinical Department of Surgical Oncology, Chair of Oncology, Medical University of Lodz between January 1998 and December 2001 were studied.

Immediately after surgery specimens for molecular study were taken from resected tumors and placed in liquid nitrogen, and next stored in -80°C. The rest of resected material was routinely fixed in 10% buffered formalin. After 24 hour fixation specimens taken from tumors were dehydrated through graded alcohols and acetones, cleared in xylenes and embedded in paraffin blocks at 56°C. For histopatological diagnosis sections 4 μm thick of formaline-fixed, paraffin embedded tissue were placed on poly-L lysine coated slides and stained with hematoxylin and eosin. All tumors were diagnosed at Department of Tumor Pathology, Chair of Oncology, Medical University of Lodz according to the WHO criteria.

All analyses were performed on archival material with the approval of the Bioethics Medical University Committee No. RNN/53/08/KE.

### DNA and RNA isolation

All analyses were performed using snap-frozen tissues stored at -80°C. DNA and RNA were co-extracted by means of Macherey-Nagel DNA/RNA purification kit from normal and tumor tissues. RNA samples were treated with DNase. RNA and DNA concentrations were measured spectrophotometrically. 100 ng of total RNA was reverse-transcribed into single-stranded cDNA in a final volume of 40 μl containing 50 mM DTT, 1.5 μg oligo(dT), 0.5 mM dNTP, 40 units of RNase inhibitor and 200 units of M-MLV reverse transcriptase (Promega).

### DNA and cDNA sequencing

Exons 5–8 of the *P53 *gene were amplified by PCR as described before and sequenced using the dideoxy termination method and SequiTherm Excel DNA Sequencing Kit (Epicentre Technologies) [1,2]. To verify the results of sequencing the semi-quantitative densitometric analysis was performed. The intensity of wild type and mutated bands was estimated comparing to the neighbouring bands in the same sequencing lane used as a reference.

### Estimation of sequencing sensitivity

DNA samples consisting of either wild type or mutated templates were obtained from control samples and specimens containing only mutated bands respectively. Moreover, a DNA sample obtained from patient with Li-Fraumeni syndrome (that we previously reported) was used as an example of a 50% presence of the mutated template [3]. Samples presenting only a mutated band (A) and 50% presence of the mutated template (B) were mixed with control samples (C) in the following proportions expressed in percent, respectively: 100/0; 87,5/12,5; 75/25; 50/50; 25/75; 12,5/87,5; 0/100 for A/C and 50/50; 40/60; 33,3/66,6; 25/75; 12,5/87,5; 0/100 for B/C.

### Immunohistochemistry

Sections 4 μm thick of formaline-fixed, paraffin embedded tissue were placed on SuperFrost Plus slides (Menzel-Glaser, Braunschweig, Germany). These were deparaffinized in xylenes and rehydrated through graded alcohols. Then, the sections were microwaved in 0.01 M sodium citrate buffer, pH 6.0, twice for 10 minutes at 360W to epitope retrieval. After the slides were rinsed in running water, washed with TRIS buffered saline, pH 7.4, and incubated for 1 hour at room temperature with the primary monoclonal antibody anti-P53 (clone DO-7, 1:100 dilution, DAKO, Glostrup, Denmark), and processed with EnVision (DAKO, Glostrup, Denmark) system. Sections were counterstained with haematoxylin, dehydrated with ethanol and cleared in xylene. For all tumors P53 labelling indices defined as the percentage of positive nuclei, were determined by counting 1000 cells in high power fields (×400). The slides were scored by two independent pathologists. When regional heterogenity of labelling was detected in the tumor, counting areas were chosen to include those with the most pronounced staining.

### Real-time quantitative RT-PCR

Real-time quantitative RT-PCR was performed on a Rotor Gene 6000 instrument (Corbett, Life Sciences, Australia) for *P53 *gene (TaqMan^® ^Gene Expression Assays no. Hs00153340_m1 and Hs00153349_m1) with *GAPDH *(TaqMan^® ^Gene Expression Assays no. Hs99999905_m1) used as a reference gene for normalization of the target genes expression levels. A normalized relative expression level for a given target gene in unknown sample versus control sample was calculated using the method described previously by Pfaffl et al. with pooled cDNA from all tumor samples used as a control, according to the equation:

where E_TARGET _and E_REF _stand for the real-time PCR efficiency of target and reference gene amplification, respectively, and ΔCP_TARGET _(control-sample) and ΔCP_REF _(control-sample) denote the difference in crossing points (CP) between unknown and control samples for a given target and reference genes, respectively [4].

### Statistical analysis

The differences in gene expression levels were evaluated by Mann-Whitney U test. Statistical significance was assumed for *P*-value ≤ 0.05.

### Loss of heterozygosity (LOH) and microsatellite instability (MSI) analyses

Analyses were performed using paired tumor specimens and control tissues to recognize hemizygous mutations of *P53 *gene by using the following microsatellite markers: D17S976, D17S675, D17S1828 and D17S729. Forward primers were 5' end fluorescence-labelled. PCR was performed in thermocycling conditions established individually for each pair of primers. The percentage of nontumor cells contaminating analyzed specimens was estimated based on LOH and MSI analysis as was already described [5].

## Results

### Sequencing of cDNA shows mutation more frequently than sequencing of DNA

**Figure 1:**
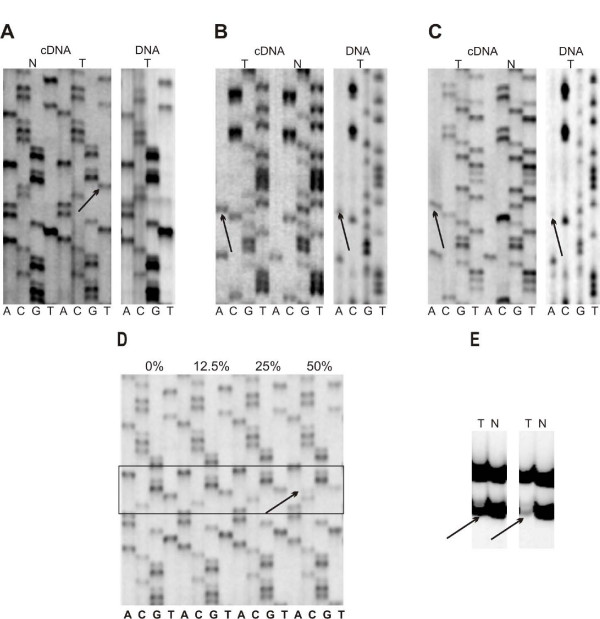
**Molecular analyses of *P53 *gene**. (**A**) cDNA sequencing showing mutation (arrow) and DNA analysis confirming presence of wild type nucleotide only (case no. 3 in Table 1). (**B**) cDNA and DNA sequencing showing both bands equally (case no. 1 in Table 1). (**C**) cDNA sequencing showing prelevance of mutated nucleotide, and DNA presenting prelevance of wild type band (case no. 9 in Table 1). (**D**) estimation of sequencing sensitivity for sample presenting only mutated band, see Materials and Methods (case no. 3 in Table 1). (**E**) example of LOH analysis for microsatellite marker D17S675. The lost allele is marked with an arrow (case no.13, left and 10, right in Table 1). LOH analysis confirms a high amount of nontumor cells (cells without LOH on 17 p) in sample no. 13. N, normal tissue (blood); T, tumor sample.

Sequencing of cDNA showed *P53 *missense mutations in 15 specimens and nonsense mutation in one case of 23 analyzed CC. DNA sequencing performed for 15 samples with missense mutation detected *P53 *gene mutation only in six samples, in another six cases, the mutated template was difficult to detect, in the final two cases the DNA analyses did not revealed a *P53 *gene alteration (for one sample DNA was not analyzed). In all cases with a *P53 *mutation, the increased ratio of mutated to wild type template was observed after cDNA analyses, when compared with DNA analyses (Figure 1, Table 1).

**Table 1:** Results of molecular and immunohistochemical analyses of *P53 *gene.

No	17 p	Mutation	Exon	Codon	Effect	cDNA	DNA	Cells without LOH 17 p	Normalized relative expression level of *P53 *mRNA
1	LOH	CGT>CAT	8	273	Arg→His	WT = MT	WT = MT	25–35%	3,201

2	LOH	GTG>ATG	5	173	Val→Met	only MT	MT>WT	15–25%	2,934

3	LOH	CGG>TGG	7	248	Arg→Trp	only MT	WT	50–60%	3,796

4	LOH	CGC>CAC	5	175	Arg→His	only MT	WT = MT	25–35%	4,235

5	LOH	CGT>CAT	8	273	Arg→His	MT>WT	WT = MT	25–35%	3,497

6	LOH	GAG>AAG	8	285	Glu→Lys	MT>WT	WT	40–50%	4,443

7	U	GGC>AGC	7	245	Gly→Ser	only MT	WT>MT	**-**	3,005

8	ROH	CGG>CAG	7	248	Arg→Gln	only MT	WT>MT	**-**	3,928

9	ROH	CGT>CAT	8	273	Arg→His	MT>WT	WT>MT	**-**	1,692

10	LOH	CGG>TGG	8	282	Arg→Trp	only MT	WT>MT	25–35%	3,307

11	ROH	GGC>AGC	7	245	Gly→Ser	MT = WT	WT>MT	**-**	2,213

12	NA	ATG>ATA	7	237	Met→Ile	only MT	NA	**-**	4,160

13	LOH	GTG>ATG	6	216	Val→Met	MT>WT	WT = MT	25–35%	2,478

14	LOH	GGC>AGC	7	245	Gly→Ser	MT = WT	WT>MT	35–45%	3,271

15	LOH	CGC>CAC	5	175	Arg→His	only MT	WT = MT	25–35%	4,215

16	LOH	negative						15–25%	2,607

17	LOH	negative						25–35%	2,932

18	LOH	negative						15–25%	2,606

19	LOH	13372 ins C	6	205	Frameshift	WT = MT	WT = MT	50–60%	1,803

20	ROH	negative						-	4,017

21	LOH	negative						15–25%	2,981

22	LOH	negative						15–25%	1,867

23	LOH	negative						25–35%	1,482

LOH, loss of heterozygosity; MT, mutated template; MT>WT, prevalence of mutated template; NA, not analyzed; WT, wild type template; WT = MT, equal amount of wild type and mutated template; WT>MT, prevalence of wild type template; ROH, retention of heterozygosity; U, uninformative (samples 1–18, positive immunohistochemistry; samples 19–23, negative immunohistochemistry).

### LOH analysis suggests presence of hemizygous P53 mutations and a high contamination of nontumor cells

Eleven of the 16 CC cases containing *P53 *mutations also showed a loss of heterozygosity on chromosome 17 p (Table 2). Two samples with a clear gene mutation, confirmed on the basis of the cDNA sequencing, but not following the DNA analysis, showed also LOH on 17 p, which attests to the presence of *P53 *hemizygous mutations. LOH analyses confirmed presence of cells without LOH at 17 p in analyzed specimens (Figure 1). The content of such cells (nontumor cells) is shown in Table 1.

**Table 2:** Summary of detailed microsatellite analysis performed for chromosome 17 p.

No	D17S1828 (3.700 kbp)	D17S675 (4.403 kbp)	D17S729 (7.156 kbp)	D17S976 (17.858 kbp)
**1**	ROH	**LOH**	**LOH**	**LOH**

**2**	**LOH**	NI	NI	**LOH**

**3**	ROH	ROH	**LOH**	NI

**4**	**LOH**	NI	NI	NI

**5**	**LOH**	NI	NI	**LOH**

**6**	**LOH**	**LOH**	**LOH**	NI

**7**	NI	NI	NI	NI

**8**	ROH	ROH	ROH	ROH

**9**	ROH	NI	NI	ROH

**10**	**LOH**	**LOH**	NI	**LOH**

**11**	ROH	NI	NI	ROH

**12**	NA	NA	NA	NA

**13**	**LOH**	**LOH**	NI	**LOH**

**14**	**LOH**	NI	**LOH**	**LOH**

**15**	NI	**LOH**	NI	**LOH**

**16**	**LOH**	NI	NI	**LOH**

**17**	**LOH**	**LOH**	NI	**LOH**

**18**	ROH	ROH	**LOH**	**LOH**

**19**	**LOH**	**LOH**	NI	**LOH**

**20**	ROH	ROH	NI	ROH

**21**	**LOH**	NI	NI	**LOH**

**22**	**LOH**	NI	NI	**LOH**

**23**	**LOH**	ROH	NI	**LOH**

LOH, loss of heterozygosity; NA, not analyzed; NI, non informative; ROH, retention of heterozygosity. Numbers in brackets indicate the distance from the centromere (kbp) determined according to HuRef Database. *P53 *(7.169 kbp) gene is located between D17S729 and D17S976.

### As much as 25% of mutated template could escape detection during DNA sequencing

The analyses of sequencing sensitivity showed the mutation following enrichment of mutated template by 25% only in one case (case no. 3 in Table 1) whereas in the second case (patient with Li-Fraumeni syndrome), the mutated template was still difficult to detect. Based on this data, we concluded that the presence of 25% of mutated template can lead to misinterpretation of sequencing results and may suggest that 25% of mutated template could be difficult to detect during sequencing of DNA (Figure 1).

### P53 mRNA is overexpressed in the subgroup of colorectal cancers if compared with normal tissues

**Figure 2:**
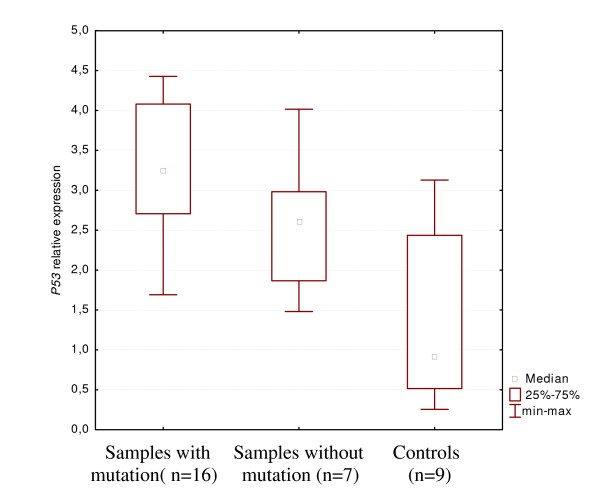
***P53 *expression level in tumor samples with mutation and tumor samples without mutation versus control tissue**. Significance of difference according to Mann-Whitney *U *test, *P *< 0.05.

*P53 *mRNA levels were compared among three groups: first, cases with *P53 *missense mutations; second, cases lacking of a *P53 *mutation, third, a control group represented by normal colon, brain and renal tissues. The differences in gene expression levels were evaluated by the Mann-Whitney U test. mRNA expression level was significantly higher (*P *= 0,0022) in the group with missense *P53 *mutation as compared with the group of control tissues. *P53 *mRNA expression was also significantly higher (*P *= 0,026) in the group of cancers without a *P53 *mutation as compared with the control group. *P53 *expression was not significantly different between two groups of cancer samples (Figure 2).

### Immunohistochemistry

**Figure 3:**
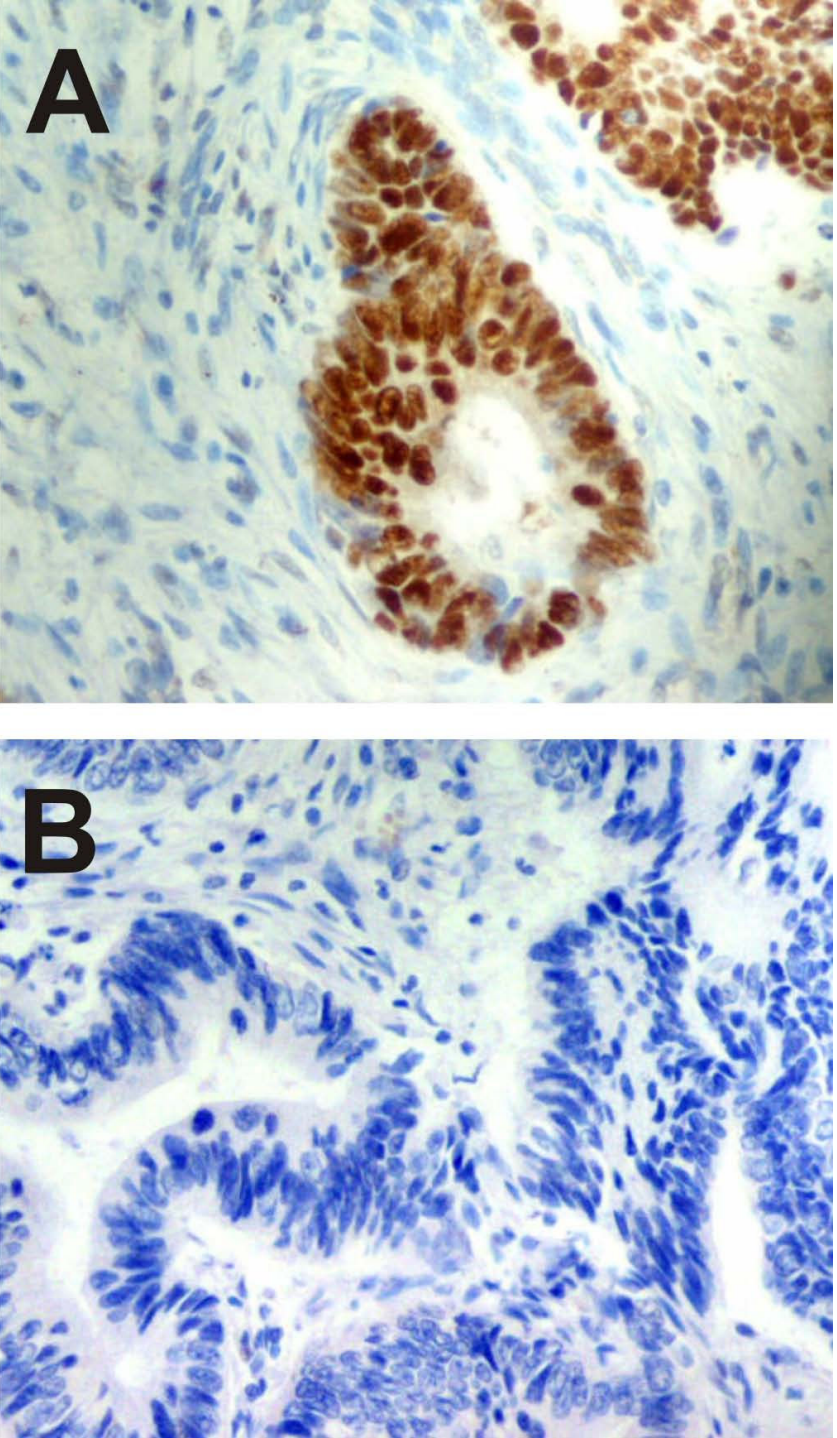
**P53 immunohistochemistry**. (**A**) case no. 2 with nuclear expression of the protein. (**B**) case no. 20 without immunopositivity for P53. Magnification 200×.

18 CC cases showed P53 positive immunoreactivity, while five cases demonstrated no immunoreactivity (Table 1, Figure 3). 15 samples with a high P53 labelling index showed missense mutations, but a single case without P53 immunoreactivity contained a nonsense mutation. *P53 *mRNA overexpression was observed in samples with both positive and negative P53 immunoreactivity.

## Discussion

We present here an analysis of colorectal cancer that supports our previous data published for glioblastoma, demonstrating that a higher amount of mutated *P53 *template is detected at the mRNA level than on the DNA level [1]. The approach based on cDNA analysis resulted in an increase of the detection of *P53 *mutations in CC. Several samples interpreted initially as ambiguous or with no *P53 *mutation on DNA sequencing, demonstrated mutations following the cDNA analysis. The explanation for this discrepancy could be the contamination of analyzed specimens by cells with the wild type *P53*, combined with mutated *P53 *mRNA overexpression, and a phenomenon of *P53 *heterozygous or hemizygous mutations. Our data supports this explanation. Histopathological examinations and LOH analysis showed a high content of normal cells in the majority of samples and the presence of hemizygous *P53 *mutations. Real time RT-PCR analyses confirmed the *P53 *mRNA overexpression in colorectal cancer cells.

Moreover it should be realized that *P53 *mutation occurs at last stage of CC tumorigenesis and is not observed in all neoplastic cells. This issue was presented by Goranova et al. and by Giaretti et al. [6,7]. Goranova et al. showed samples presenting: neoplastic cells with *P53 *mutation, and cells showing *APC *and/or *K-Ras *mutation but not *P53 *mutation [6]. Contamination or enrichment of tumor samples by cells without *P53 *mutation (normal and/or neoplastic) is obviously causing difficulties in *P53 *mutation detection during colorectal cancer DNA analysis. In addition similar discrepancies between *P53 *mRNA and DNA analyses for breast cancers were already published by Williams et al. [8].

Heterozygous or hemizygous mutations of *P53 *have been observed frequently [9,10]. Disregard of the latter phenomenon appears questionable in the light of data presented here. If a tumor sample contains 50% of tumor cells with heterozygous mutation, only 25% of mutated template can be detected. Similarly, a sample containing a hemizygous mutation will show 33% of mutated template. We showed here that the mutated template present in the range of 25% to 30% could be really difficult to detect during sequencing analysis. Analogous data of the sequencing sensitivity have been already published by Cheng and Haas [11].

Since contamination is certain and mRNA analysis prevails DNA analysis, overexpression of mutated *P53 *mRNA has to be accepted as logical. Our results showing dominance of mutated template following the cDNA sequencing and an almost complete absence of mutated template following the DNA analysis could not be explained only by the elimination of wild type allele at the mRNA level. In contrast, overexpression of mutated mRNA is suggested by our analysis. However, we still cannot exclude that upregulation of *P53 *mutated allele coexists with silencing of the wild type allele in colorectal cancer. Overexpression of *P53 *mRNA occurring both in cancer with and without *P53 *mutation may result from *P53 *gene activation by DNA damage, *K-Ras *activation, etc.

It must be stressed that the explanation of discrepancies between the DNA and the cDNA analyses detected in CC cannot be generalized for other cancers. Colorectal cancers most likely carry hemizygous mutations, whereas we have already found that glioblastomas contain mostly heterozygous mutations of *P53*. Explanatory conditions for discrepancies between direct sequencing of the cDNA and the DNA analyses could be applied without any additional factors only for those cases presenting hemizygous mutations of *P53*. However, cases presenting the heterozygous mutation require searching for mechanisms responsible for selective overproduction of mutated *P53 *mRNA only. Nevertheless, from hypotheses presented for glioblastomas, the one suggesting selective overproduction of mutated *P53 *seems to be most relevant to CC [1].

Our data has also shed new light on the P53 protein accumulation in the context of *P53 *mutation. A correlation between *P53 *mutations and the accumulation of P53 in the nucleus has been observed a long time ago [12,13]. Furthermore, discrepancies between the immunohistochemical and the DNA molecular analyses, analogous to those observed by us, have also been presented many times. They were always attributed to the decreasing value of immunohistochemistry [14-16]. Our data based on the sequencing of both DNA and cDNA and, in addition, immunohistochemistry did not suggest that the DNA molecular analysis is always more robust to detect *P53 *mutation in CC. We suggest that cellular heterogeneity could at least partially explain, the discrepancies observed during the P53 immunohistochemical analysis and the *P53 *DNA sequencing in favor of immunohistochemistry. However one should be aware of the fact that already published correlations and discrepancies between P53 accumulation and *P53 *mutations, were analyzed in many experimental conditions. SSCP (single strand conformation polimorphism) analysis, DHPLC (denaturing high performance liquid chromatography) technique, direct DNA sequencing, presence of missense and nonsense mutaitons, etc. All these factors biase interpetation of data, dedicated to compare immunohistochemical and molecular analysis of P53/*P53*. We do not intend to oversimplify the very complex issue of correlations between immunohistochemical and molecular analysis. Nevertheless our new approach based on the cDNA analysis increases *P53 *mutation detectability and allows to decrease the number of CC cases presenting abundant P53 nuclear accumulation but lacking the *P53 *mutation. Our data suggests that *P53 *mutations detection in colorectal cancer can be improved. We are aware that techniques such as DHPLC allowing to collect the mutant peaks by means of fraction collection increases sensitivity of the nucleic acid mutation detection. However it should be remembered that this technique although very sensitive, in terms of the percentage of mutated template detected, does not allow to detect all kinds of mutations [17-20]. To this end we recomend to increase sensitivity of missense mutations detection by direct cDNA sequencing. *P53 *cDNA DHPLC followed by sequencing of collected mutated template should also be considered. Detection of nonsense mutations has to be done by DNA analysis, since decay of nonsense mutated mRNA, will negatively influence sensitivity of cDNA analysis.

We would like to add that the observed overexpression of *P53 *mRNA in colorectal cancer cells does not seem to be responsible for P53 protein accumulation. Almost all CC samples showed the *P53 *mRNA overexpression, including samples with no P53 nuclear accumulation.

## Conclusion

The most important findings of this study are as follows: 1, cDNA sequencing appears superior to DNA sequencing in detecting *P53 *missense mutations in colorectal cancer; 2, colorectal cancer demonstrates overexpression of mutated *P53 *mRNA; 3, positive P53 immunoreactivity in CC, along with negative results for *P53 *DNA sequencing, should not be so readily discarded in favor of the results of DNA analysis [16,21]. We suggest that the *P53 *cDNA sequencing should be applied for all cancers, since *P53 *mRNA analysis can increase mutation detectability.

## Competing interests

The authors declare that they have no competing interests.

## Authors' contributions

MS, MZ and PR designed project and performed sequencing and MSI analyses. PR and PPL was responsible for supervising and founding acquisition. DKW and IZ performed LOH analyses. RK, GPW and DJK participated in providing samples for experiments and in obtaining immunohistochemical data. RS performed Real-time PCR and statistical analysis. All authors participated in analysis and interpretation of obtained data. All authors have been involved in drafting the manuscript. All authors have given approval of the final version of the manuscript.

## Pre-publication history

The pre-publication history for this paper can be accessed here:

http://www.biomedcentral.com/1471-2407/9/278/prepub
